# Infantile Sandhoff disease with ventricular septal defect: a case report

**DOI:** 10.1186/s13256-022-03550-0

**Published:** 2022-08-25

**Authors:** Jamal Khaled Sahyouni, Luma Bassam Mahmoud Odeh, Fahad Mulla, Sana Junaid, Subhranshu Sekhar Kar, Naheel Mohammad Jumah Al Boot Almarri

**Affiliations:** 1 Department of Paediatrics, RAK College of Medical Sciences, RAK Medical and Health Sciences University, Ras Al Khaimah, United Arab Emirates; 2 Department of Paediatrics, RAK College of Medical Sciences, RAK Medical and Health Sciences University, Ras Al Khaimah, United Arab Emirates; 3 Saqr Hospital, Ras Al Khaimah, United Arab Emirates; 4 Adjunct Clinical Faculty, RAK College of Medical Sciences, RAK Medical and Health Sciences University, Ras Al Khaimah, United Arab Emirates

**Keywords:** Infantile Sandhoff disease, Ventricular septal defect, Hexosaminidase, Neuroregression, Cherry-red spots, Hypotonia

## Abstract

**Background:**

Infantile Sandhoff disease is a rare inherited disorder that progressively destroys nerve cells in the brain and spinal cord, and is classified under lysosomal storage disorder. It is an autosomal recessive disorder of sphingolipid metabolism that results from deficiency of the lysosomal enzymes β-hexosaminidase A and B. The resultant accumulation of GM2 ganglioside within both gray matter nuclei and myelin sheaths of the white matter results in eventual severe neuronal dysfunction and neurodegeneration.

**Case presentation:**

We evaluated a 3.5-year-old Comorian girl from the United Arab Emirates who presented with repeated chest infections with heart failure due to ventricular septal defect, neuroregression, recurrent seizures, and cherry-red spots over macula. She had macrocephaly, axial hypotonia, hyperacusis, and gastroesophageal reflux. Organomegaly was absent. Brain magnetic resonance imaging, metabolic tests, and genetic mutations confirmed the diagnosis. Despite multidisciplinary therapy, the girl succumbed to her illness.

**Conclusion:**

Though early cardiac involvement can be seen with novel mutations, it is extremely rare to find association of ventricular septal defect in infantile Sandhoff disease. Neuroregression typically starts around 6 months of age. We report this case because of the unusual association of a congenital heart disease with underlying infantile Sandhoff disease and symptomatic heart failure in the first month of life with eventual fatal outcome.

## Background

Lysosomal storage disorders (LSDs) are a specific group of inborn errors of metabolism including more than 50 different diseases caused by a structural defect or deficiency of lysosomal enzymes [1]. GM2 gangliosidosis is one group of LSD that is classified into three types: Sandhoff disease (SD), Tay–Sachs, and GM2 activator deficiency (GM2A-AB variant) [2]. SD is a rare autosomal recessive neurodegenerative disorder prevalent 1 in 384,000 live births, related to a genetic deficiency of the enzyme β-hexosaminidase (HEX) [1]. There are two active forms of this enzyme—β-HEX A, composed of one α and one β subunit (αβ), and β-HEX B, which contains two β subunits (ββ). In SD, there is a mutation in HEX B gene that encodes the α subunit, thereby leading to β-HEX enzyme deficiency [2]. Tay–Sachs disease, SD, and GM2AP deficiency are caused by biallelic pathogenic variants of human HEXA, HEXB, and GM2AP gene in respective order [3].

Gangliosides have been found within the gray matter nuclei and myelin sheaths of the white matter [4]. When GM2 gangliosides accumulate within lysosomes of cortical neurons, distension of neuronal cell bodies with displacement of nucleus occurs, which leads to cellular enlargement and severe neurodegeneration [4–9]. There is evidence of demyelination as well as delayed myelination, which can be attributed to gray matter disease [10–12]. Autopsy findings are suggestive of lipid storage and edema formation in white matter [13].

Described by Warren Tay in 1881, the clinical manifestation can be divided into three forms based on age group: infantile, juvenile, and adult [14]. Infantile SD presents with truncal hypotonia, muscle weakness, hyperacusis, developmental delay and regression, seizure, and cherry-red spots on ophthalmologic examination around 6 months of age [15]. Hepatosplenomegaly, coarse facies, and bone abnormality are seen less often than Tay–Sachs disease [14]. Death occurs by 3 years of age owing to intractable seizure and aspiration pneumonia. Though late cardiac involvement is reported with cardiomegaly and heart murmur in SD, association of ventricular septal defect has not been reported in literature [16]. This rare case is reported for the purpose of acquaintance with clinical symptomatology, recognizing unusual association of congenital cardiac disease, identifying other comorbidities, and interpreting abnormal neuroradiology.

## Case presentation

We evaluated a Comorian girl aged 3 years 6 months with neuroregression and seizures. The child was third born to second-degree consanguineous parents by lower-segment cesarean section due to cephalopelvic disproportion. Birth weight was 3.1 kg with uneventful perinatal history. There was maternal history of normal healthy live birth in the first pregnancy and spontaneous miscarriages in second and fourth pregnancies in early trimesters. The fourth pregnancy was associated with Down syndrome. The mother was given antenatal progesterone for excess bleeding in the first trimester.

**Fig. 1:**
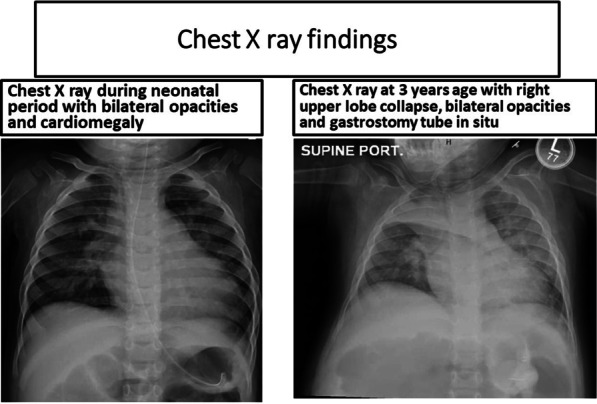
Chest X-ray findings

**Fig. 2:**
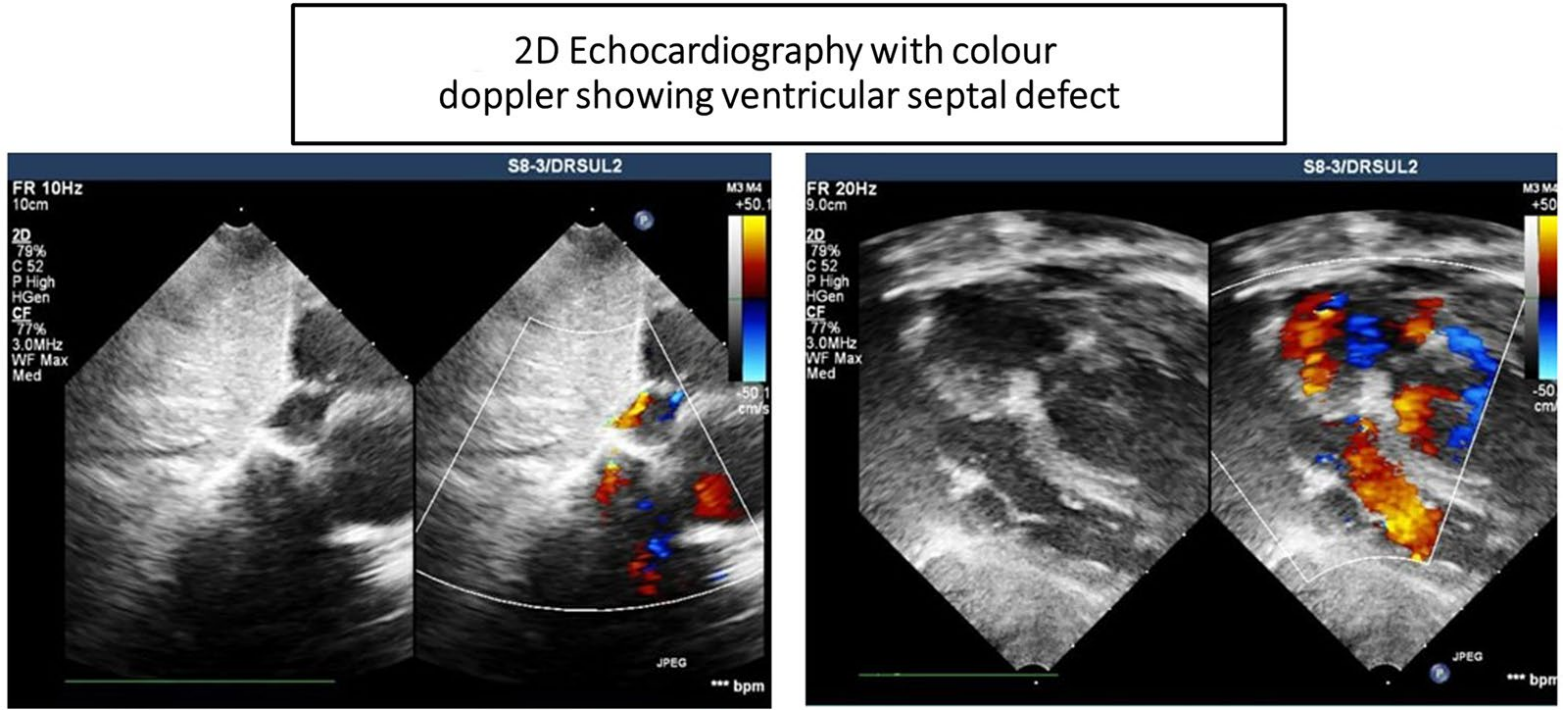
Two-dimensional echocardiography with color Doppler showing ventricular septal defect

From 2 weeks of age, she presented with lethargy, sweating, and breathlessness on feeding. Later on, she presented with recurrent episodes of aspirations with severe lower respiratory infections. Cardiac examination revealed a holosystolic murmur suggestive of ventricular septal defect (VSD). Chest X-ray revealed cardiomegaly with features suggestive of pneumonia (Fig. 1). Echocardiography (ECHO) showed moderate VSD (6–7 cm subaortic perimembranous VSD), dilated left atrium and left ventricle, trivial aortic regurgitation with aortic cusp collapse, dilated pulmonary artery system with flow acceleration across pulmonary valve, and half-systemic pulmonary artery pressure with normal left ventricular systolic function (Fig. 2). There was no pericardial effusion or right ventricular outflow tract obstruction. She was managed medically with decongestive medications and antibiotics for lower respiratory infections. She was noted to have laryngomalacia. At 3 months of age, decreased motor movements were noted. She was gaining weight till 5 months of age, after which there was flattening of the growth curve and failure to thrive. At 6 months, she had developmental arrest followed by progressive neuroregression. She also had severe startle response since 8 months of age. Then, she started having generalized recurrent seizures from 9 months onward. The epileptic episodes were mostly focal with secondary generalization, with the most severe event reported as having frequency of ten seizure episodes within 2 hours time period despite anticonvulsant therapy. She had also macrocephaly with coarse facial features, persistent laryngomalacia, and hyperacusis. There was no muscle atrophy. Central hypotonia, peripheral hypertonia, and a positive Babinski reflex were elicited. Organomegaly was absent. Ophthalmological examination showed bilateral macular cherry-red spots and an inability to fixate the eyes. At 12 months, she developed gastroesophageal reflux disease (GERD) as well as reactive airway disease. Gastrostomy tube feeding was also commenced. She had frequent episodes of hospitalizations due to repeated aspiration pneumonia, reactive airway diseases, and other central nervous system complications.

**Fig. 3:**
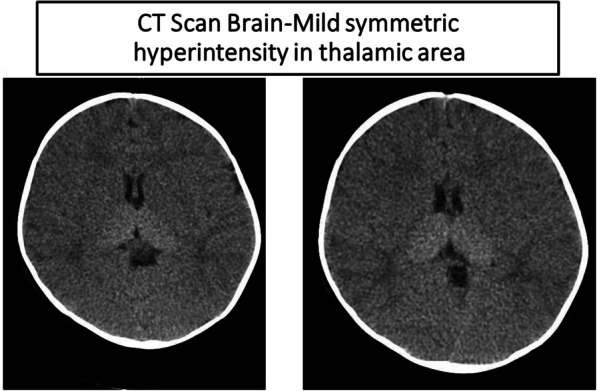
Computerized tomography scan of brain showing mild symmetric hyperintensity in thalamic area

**Fig. 4:**
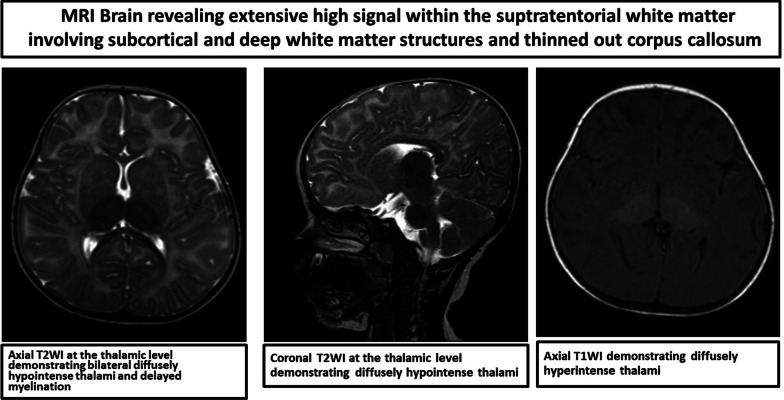
Magnetic resonance imaging of brain revealing extensive high signal within the supratentorial white matter involving subcortical and deep white matter structures and thinned-out corpus callosum

History and physical examinations pointed toward the diagnosis of GM2 gangliosidosis (Tay–Sachs disease, SD, AB variant). In view of cherry-red spots and coarse facies, GM1 gangliosidosis was also considered. No significant abnormality was noted in complete blood count, electrolytes, or renal and liver function tests. Ultrasonography of abdomen did not reveal any hydronephrosis or other anatomic abnormalities. Computerized tomography scan of brain without contrast was suggestive of mild bilateral symmetric hyperdensity of thalami (Fig. 3). Electroencephalogram (EEG) showed slowing of delta frequencies associated with drowsiness. Video-fluoroscopic assessment for swallowing function was suggestive of aspiration on both fluoroscopic runs. Magnetic resonance imaging (MRI) of brain revealed extensive high signal within the supratentorial white matter involving subcortical and deep white matter structures. There was evidence of T1-increased signal in the thalamus and a relatively large head shape. Bilaterally, the thalami demonstrated symmetric reduction of T2 signal and increase in the T1-weighted signal. There was marked delay in myelination as demonstrated on T1-weighted imaging. The corpus callosum was markedly thinned in its anterior body and genu. There was mild hypoplasia of the posterior arch of the C1 vertebra causing minimal narrowing at the upper cervical spinal canal (Fig. 4).

**Fig. 5:**
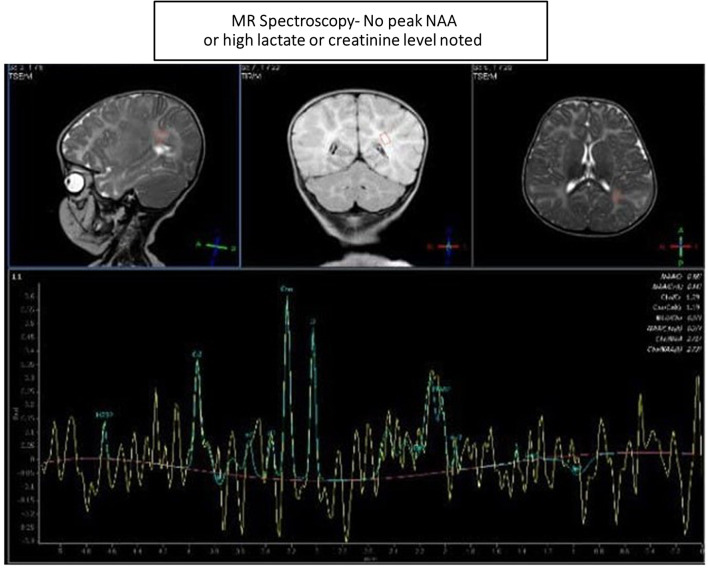
Magnetic resonance spectroscopy showing no peak *N*-acetyl aspartate or high lactate or creatinine level

Magnetic resonance spectroscopy (MRS) trace did not reveal high creatinine or *N*-acetyl aspartate (NAA) peaks. No significant lactate level was demonstrated (Fig. 5).

Metabolic workup revealed a serum finding of trace-to-absent total serum HEX A and HEX B (0.0 nmol/min/ml; reference value > 20 nmol/min/ml) explaining the deficiency of the β subunit of HEX and consequent deficiency of HEX B. The serum HEX A percentage was 100% (reference value 20–90%). This biochemical findings of low total HEX and deficient HEX B activities, with high percentage of HEX A/total HEX activity suggested the diagnosis of SD. Oligosaccharide urine screen was positive in the urine sample, and genetic testing confirmed the diagnosis of SD with homozygous deletion c.(445+1_512-1)_(669+1_1170) in the HEXB gene. The parents were advised to consent to genetic analysis, but they refused.

**Fig. 6:**
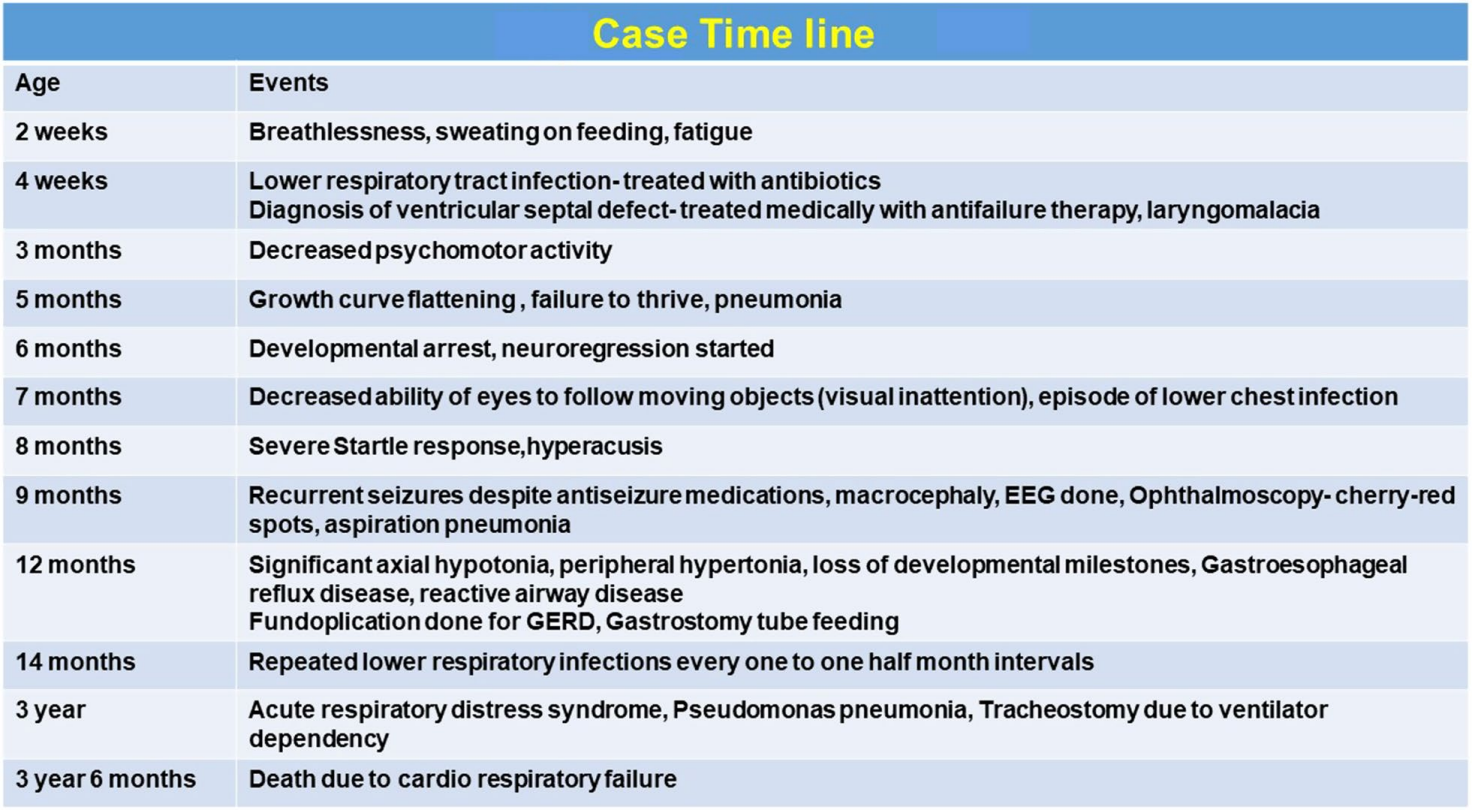
Case timeline

The patient was maintained on decongestive therapy (captopril, frusemide, spironolactone, and digoxin) and antiepileptics (levetiracetam and phenobarbitone). Fundoplication was done owing to her symptomatic GERD during infancy, and she was started on regular esomeprazole and domperidone, after which she was fed through gastrostomy tube. Fluticasone, ipratropium bromide and salbutamol nebulizations were continued in view of reactive airway disease. Iron supplementation was started in view of anemia. The clinical course is complicated with recurrent aspiration pneumonia warranting frequent hospital admissions. She also underwent multiple bronchoscopies. At 3 years of age, she had adenoviral infection on respiratory BioFire assay and then developed *Pseudomonas* pneumonia. Despite treatment with piperacillin–tazobactam, ciprofloxacin, tobramycin, and clindamycin antibiotics, her cardiorespiratory status worsened and she became ventilator dependent. Tracheostomy was performed at 3 years of age. However, despite the multimodality care with cardiology, neurology, pulmonology, physiotherapy, and nutritional and ventilatory support, she died at 3 and half years of age (Fig. 6).

## Discussion and conclusions

SD is an autosomal recessive lysosomal storage disorder on chromosome 5q13 and was first described by Konrad Sandhoff in 1968. It is due to hexosaminidase A and B enzyme deficiency (due to an abnormal β subunit) leading to accumulation of glycosphingolipids in neuronal cells and subsequent neurodegeneration [14]. The juvenile form of SD is present between 2 and 10 years of age with dysarthria, ataxia, mental deterioration, and seizures. Organomegaly and cherry-red spots are uncommon. The adult form of SD is characterized by movement disorder, pyramidal and extrapyramidal signs and symptoms of lower motor neuron disease, and supranuclear ophthalmoplegia [14]. Progressive systemic accumulation of sphingolipids leads to macrocephaly, cherry-red spots in the eye, skeletal dysostosis, and organomegaly [17].

In a study of infantile-onset Sandhoff disease in 2018 in Iran, involving 25 patients with the condition, the most common and earliest clinical manifestations were motor and cognitive milestones delay and regression [2]. Cherry-red spots are a characteristic ocular and physical sign of infantile SD and can be used for early detection in patients who are suspected to have this disease. Organomegaly was detected in only two patients, and some associations between diffuse Mongolian spots and SD were found to be significant [2]. Accumulation of calcium associated with collection of GM2 ganglioside leads to gliosis and loss of myelin and axon in cortical neurons, giving rise to some of the earliest findings on T2-weighted images of brain MRI, including bilateral thalamic hypodensity and hypomyelination, that are characteristic of brain involvement in the infantile SD disease, which were detected in the same study [7, 18]. An increase in a specific marker *N*-acetylhexosamine at 2.07 ppm in white matter and thalamus on proton MR spectroscopy of patients with SD was also reported, in addition to the mentioned finding [15, 19]. Diagnosis is confirmed by enzymatic assays revealing a deficiency of both lysosomal hydrolase β-HEX A and β-HEX B. Decreased levels of HEX A and HEX B are seen in patients with SD but solitarily decreased levels of HEX A are seen in Tay–Sachs disease [19]. Genetic study is important to make definitive diagnosis and to help family planning and prenatal diagnosis in affected families [2]. As there is a high rate of association with consanguineous marriages and SD seen in several studies, it is very important that all couples be advised to undergo premarital genetic testing and counseling for any carrier genes.

Though cardiac involvement has been reported in an 18-month-old boy alongside the classical neurological features, the child exhibited severe mitral regurgitation secondary to mitral valve prolapse and mild aortic regurgitation from aortic valve prolapse. He also had asymmetric hypertrophy of the interventricular septum without left ventricular outflow tract obstruction [16]. Similarly, another case report describes a 14-month-old female baby who exhibited mitral regurgitation and cardiomegaly at the age of 2 months and dilation of the left atrium and left ventricle at the age of 6 months with SD [20] However, congenital heart disease such as ventricular septal defect has not yet been reported in infantile SD. The association of VSD with Sandhoff disease is likely coincidental, and there is no pathophysiological mechanism to explain this association.

The disease progresses rapidly, with deaths occurring by 3–5 years of age. Case fatality in the infantile form typically occurs before the age of 4 owing to extensive and severe central nervous deterioration [21]. For the treatment of SD, studies are still being conducted worldwide, and a definitive treatment other than supportive measures has not yet been recommended. One ongoing study in Tehran, Iran is showing efficacy of miglustat therapy in SD, but miglustat is not yet an approved drug for this condition [22].

In conclusion, infantile SD is an important differential diagnosis for each child presenting with neurologic symptoms such as developmental delay, neuroregression, and cherry-red spots on ophthalmic examination. Organomegaly is not a frequent clinical finding in infantile SD. There is genetic heterogeneity among patients with SD. Early cardiac involvement is rare. We report this case because of its rare association with acyanotic congenital heart disease and to emphasize the importance of proper genetic counseling.

## Data Availability

All data generated or analyzed during this study are included in this published article.

## Abbreviations

LSDs: Lysosomal storage disorders
SD: Sandhoff disease
HEX: Hexosaminidase
VSD: Ventricular septal defect
ECHO: Echocardiography
EEG: Electroencephalogram
GERD: Gastroesophageal reflux disease
MRI: Magnetic resonance imaging
MRS: Magnetic resonance spectroscopy
NAA: *N*-acetyl aspartate

## References

[1] Maegawa GHB, Stockley T, Tropak M, Banwell B, Blaser S, Kok F, *et al*. The natural history of juvenile or subacute GM2 gangliosidosis: 21 new cases and literature review of 134 previously reported. Pediatrics. 2006;118(5):1550–62. 10.1542/peds.2006-0588. PMC2910078 17015493

[2] Tavasoli AR, Parvaneh N, Ashrafi MR, Rezaei Z, Zschocke J, Rostami P. Clinical presentation and outcome in infantile Sandhoff disease: a case series of 25 patients from Iranian neurometabolic bioregistry with five novel mutations. Orphanet J Rare Dis. 2018;13(1):130. 10.1186/s13023-018-0876-5. PMC6091055 30075786

[3] Mahuran DJ. Biochemical consequences of mutations causing the GM2 gangliosidoses. Biochim Biophys Acta. 1999;1455(2–3):105–38. 10.1016/s0925-4439(99)00074-5. 10571007

[4] Koelfen W, Freund M, Jaschke W, *et al*. GM-2 gangliosidosis (Sandhoff’s disease): two year follow-up by MRI. Neuroradiology. 1994;36:152–4. 10.1007/BF00588086. 8183460

[5] Brismar J, Brismar G, Coates R, *et al*. Increased density of the thalamus on CT scans in patients with GM2 gangliosidoses. Am J Neuroradiol. 1990;11:125–30. PMC8332498 2105593

[6] Chen C-Y, Zimmerman RA, Lee C-C, *et al*. Neuroimaging findings in late infantile GM1 gangliosidosis. Am J Neuroradiol. 1998;19:1628–30. PMC8337479 9802482

[7] Autti T, Joensuu R, Aberg L. Decreased T2 signal in the thalami may be a sign of lysosomal storage disease. Neuroradiology. 2007;49(7):571–8. 10.1007/s00234-007-0220-6. 17334752

[8] Becker LE. Lysosomes, peroxisomes and mitochondria: function and disorder. Am J Neuroradiol. 1992;13:609–20. PMC8333218 1566721

[9] Lowe JP, Stuckey DJ, Awan FR, *et al*. MRS reveals additional hexose *N*-acetyl resonances in the brain of a mouse model for Sandhoff disease. NMR Biomed. 2005;18:517–26. 10.1002/nbm.984. 16206131

[10] Alkan A, Kutlu R, Yakinci C, *et al*. Infantile Sandhoff’s disease: multivoxel magnetic resonance spectroscopy findings. J Child Neurol. 2003;18:425–8. 10.1177/08830738030180061201. 12886980

[11] Assadi M, Baseman S, Janson C, *et al*. Serial 1H-MRS in GM2 gangliosidoses. Eur J Pediatr. 2007. 10.1007/s00431-007-0469-0. 17387512

[12] Kroll RA, Pagel MA, Roman-Goldstein S, *et al*. White matter changes associated with feline GM2 gangliosidosis (Sandhoff disease): correlation of MR findings with pathologic and ultrastructural abnormalities. Am J Neuroradiol. 1995;16:1219–26. PMC8337836 7677013

[13] Huang J, Trasler JM, Igdoura S, *et al*. Apoptotic cell death in mouse models of GM2 gangliosidosis and observations on human Tay–Sachs and Sandhoff disease. Hum Mol Genet. 1997;6(11):1879–85. 10.1093/hmg/6.11.1879. 9302266

[14] Muralidharan CG, Tomar RP. Infantile Sandhoff disease: unusual presentation. Med J Armed Forces India. 2016;72(Suppl 1):S91–3. 10.1016/j.mjafi.2015.11.008. PMC5192172 28050081

[15] Saouab R, Mahi M, Abilkacem R, Boumdin H, Chaouir S, Agader O, *et al*. A case report of Sandhoff disease. Clin Neuroradiol. 2011;21(2):83–5. 10.1007/s00062-010-0035-4. PMC3145082 21153386

[16] Venugopalan P, Joshi SN. Cardiac involvement in infantile Sandhoff disease. J Paediatr Child Health. 2002;38(1):98–100. 10.1046/j.1440-1754.2002.00765.x. 11869411

[17] Kumar D, Ramanathan S, Khanna M, *et al*. Bithalamic T2 hypointensity: a diagnostic clue for Sandhoff disease. Neurol India. 2014;62:481–2. 10.4103/0028-3886.141311. 25237979

[18] Lakshmi S, Fatima Shirly Anitha G, Vinoth S. A rare case of Sandhoff disease: two in the same family. Int J Contemp Pediatr. 2015;2(1):42–5. 10.5455/2349-3291.ijcp20150210.

[19] Karimzadeh P, Jafari N, Nejad Biglari H, Jabbeh Dari S, Ahmad Abadi F, Alaee MR, Nemati H, Saket S, *et al*. GM2-gangliosidosis (Sandhoff and Tay Sachs disease): diagnosis and neuroimaging findings (an Iranian pediatric case series). Iran J Child Neurol. 2014;8(3):55–60. PMC4135282 25143775

[20] Lee HF, Chi CS, Tsai CR. Early cardiac involvement in an infantile Sandhoff disease case with novel mutations. Brain Dev. 2017;39(2):171–6. 10.1016/j.braindev.2016.09.006. 27697305

[21] Der Kaloustian VM, Khoury MJ, Hallal R, *et al*. Sandhoff disease: a prevalent form of infantile GM2 gangliosidosis in Lebanon. Am J Hum Genet. 1981;33:85–9. PMC1684873 7468596

[22] Effects of miglustat therapy on infantile type of Sandhoff and Taysachs diseases (EMTISTD) (EMTISTD).2021 ClinicalTrials.gov Identifier: NCT03822013URL:https://clinicaltrials.gov/show/NCT03822013Accessed 17 September 2021.

